# Cure of Troubles

**Published:** 1858-07

**Authors:** 


### CURE OF TROUBLES.

An unknown correspondent sends us the following, which, we judge, was written by one well on in years, who has had his full share of sorrow. We trust his last days may be his best days, and that he may go down to his "grave, like as a shock of corn cometh in his season."

Dr. W. W. Hall—Dear Sir: When a man performs a good act from disinterested motives, or confers a favor without hope or expectation of reward, other than springs from the inevitable laws of our being—for our beneficent Creator has so formed us that "virtue is ever its own reward"—when, I say, any one does such an act, all good people will approve him, and those benefited ought to acknowledge the favor, and thank him. If this was more generally done, I conceive that there would be more kindness in this world of ours; for, if gratitude is not manifested, a strong incentive to goodness is absent. It is upon this principle that I pen these few lines. I wish to thank you for myself, and in behalf of every suffering son and daughter of affliction, to thank you cordially, and from the heart, for the brief and comprehensive article in your June number, entitled "*Leaning on Providence.*" I would rather be the author of that short paragraph than to have written "Paradise Lost," or "Macbeth," or "Hamlet." To how many drooping, sorrowing hearts will it bring life and comfort? How many will receive new energy and resolution to "suffer and sin not," from its perusal? How many crushed and almost withered hopes will revive under its cheering influence? To how many breasts now filled with withering care and cankering anxiety will it bring repose and peace? And—my hand trembles while I trace the thought—how many hands that were being raised in frantic madness to summarily end a wretched existence, will be palsied by its Christian spirit of love and sympathy? Eternity will solve these questions. For one, I can say, that dark and unusual thoughts had, for days and nights, been intruding themselves upon me; and if, in my case, the effort to expel them was severe, how hard must be the struggle in the case of those still more wretched? I thank God for that little paragraph; and, next to Him, I thank *you*, sir. May His choicest blessings rest upon you My heart is full, and I could write for hours; but as I am intruding upon your attention unsolicited. I will close.

Affectionately yours,

